# T180. LOWER GLUTAMATE LEVEL IN TEMPORO-PARIETAL AREA MAY PREDICT A BETTER RESPONSE TO TDCS IN SCHIZOPHRENIA: A PILOT STUDY

**DOI:** 10.1093/schbul/sby016.456

**Published:** 2018-04-01

**Authors:** Junhee Lee, Youngwoo Yoon, Andrea Wijtenburg, Laura Rowland, In Chan Song, Kang Ik Cho, Minah Kim, Tae Young Lee, Jun Soo Kwon

**Affiliations:** 1 Seoul National University Hospital; 2 Institute of Human Behavioral Medicine, SNU-MRC; 3 Maryland Psychiatric Research Center; 4 Seoul National University College of Medicine

## Abstract

**Background:**

Transcranial Direct Current Stimulation (tDCS) is a non-invasive neuromodulation technique which uses a weak electric current from electrodes across the scalp to modulate targeted brain areas. It has been suggested that tDCS may be useful in reducing psychotic symptoms such as auditory hallucination. The aim of this study was to find alteration of key neurotransmitters in schizophrenia in temporo-parietal area (TPA) after tDCS intervention, using magnetic resonance spectroscopy (MRS) technique.

**Methods:**

Ten schizophrenia patients with auditory hallucination were recruited from the outpatient clinic of Seoul National University Hospital (SNUH). The anode was placed over the left dorsolateral prefrontal cortex (DLPFC), and the cathode was placed over the left TPA. Patients underwent MRS scan with the very short echo time phase rotation STEAM sequence before and after the tDCS sessions, respectively.

**Results:**

Seven of the participants completed MRS scans before and after the tDCS sessions. Positive and Negative Symptom Scale (PANSS) total and general psychophathology scale showed a significant improvement after tDCS. There was no significant difference between glutamate/creatinine (Cr) level before and after tDCS sessions. However, a significant positive correlation between the pre-tDCS glutamate/Cr value in left TPA and the improvement in auditory hallucination measured by Auditory Hallucination Rating Scale (AHRS) after tDCS was found.

**Discussion:**

The results of this investigation show that the schizophrenia patients whose auditory hallucination benefits the most from tDCS treatment had lower glutamate/Cr level in left TPA. Previous studies regarding the relationship between glutamatergic system and treatment response mostly have only focused on the frontal area and striatum. However, this study suggests a potential role of glutamatergic system in TPA in predicting treatment response of auditory hallucination.

